# MBL Interferes with Endovascular Trophoblast Invasion in Pre-Eclampsia

**DOI:** 10.1155/2012/484321

**Published:** 2011-12-06

**Authors:** Chiara Agostinis, Fleur Bossi, Elisa Masat, Oriano Radillo, Maddalena Tonon, Francesco De Seta, Francesco Tedesco, Roberta Bulla

**Affiliations:** ^1^Institute for Maternal and Child Health, IRCCS “Burlo Garofolo”, 34137 Trieste, Italy; ^2^Department of Life Sciences, University of Trieste, Via Valerio 28, 34127 Trieste, Italy

## Abstract

The spiral arteries undergo physiologic changes during pregnancy, and the failure of this process may lead to a spectrum of pregnancy disorders, including pre-eclampsia. Our recent data indicate that decidual endothelial cells (DECs), covering the inner side of the spiral arteries, acquire the ability to synthesize C1q, which acts as a link between endovascular trophoblast and DECs favouring the process of vascular remodelling. In this study, we have shown that sera obtained from pre-eclamptic patients strongly inhibit the interaction between extravillous trophoblast (EVT) and DECs, preventing endovascular invasion of trophoblast cells. We further demonstrated that mannose-binding lectin (MBL), one of the factor increased in pre-eclamptic patient sera, strongly inhibits the interaction of EVT with C1q interfering with the process of EVT adhesion to and migration through DECs. These data suggest that the increased level of MBL in pre-eclampsia may contribute to the failure of the endovascular invasion of trophoblast cells.

## 1. Introduction

The decidua is a newly formed tissue on the maternal side of human placenta and is characterized by active angiogenesis and structural modifications of the spiral arteries in the early phase of pregnancy. These changes, that include gradual loss of the musculoelastic structure of the arterial wall and replacement by amorphous fibrinoid material, are essential to create vessels of low resistance unresponsive to vasoconstrictive agents [1, 2] allowing continuous blood flow in the intervillous space. 

An additional feature of the physiologic changes of spiral arteries is the endovascular invasion of extravillous trophoblast (EVT) that adheres to and replaces endothelial cells (ECs) giving rise to mosaic vessels in which trophoblast and ECs coexist [3]. Recently, we have provided data indicating that decidual endothelial cells (DECs) lining the spiral arteries acquire the ability to synthesize C1q. This protein binds avidly to the cell surface and acts as a physical link between endovascular trophoblast and DECs favouring the process of vascular remodelling [4]. C1q is a recognition molecules of the complement (C) system, one of the major components of humoral innate immunity, acting as a first line of defence against microbes. The C system can be activated via three pathways, namely, the classical, the alternative, and the lectin pathway, which are triggered by the three recognition molecules, C1q, C3, and mannose-binding lectin (MBL), respectively [5]. The system is also involved in the elimination of dead or modified self cells [6], but new roles in inflammatory, immunological processes, and tissue remodelling are emerging.

Failure of spiral artery to undergo transformation may lead to a spectrum of pregnancy disorders, including pre-eclampsia [7], foetal growth restriction, and miscarriage [8, 9]. Pre-eclampsia is a complication of pregnancy characterized by hypertension and proteinuria and develops in normotensive pregnant women after midgestation. Inflammation and innate immunity seem to play an important role in the aetiology of pre-eclampsia [10]. Several recent studies suggest an association between increased complement dysregulation and pre-eclampsia [11]. The role of the lectin pathway in the onset of this syndrome is a controversial issue. The activity of MBL-MBL associated serin proteases (MASP)2 complexes is not increased in pre-eclamptic (PE) women [12]. A higher concentration of MBL has been demonstrated in the plasma of patients, compared to normal pregnant women [13] although the functions of this molecule in pregnancy remains to be clarified, despite the increased serum MBL concentration during pregnancy [14]. The association of a genetically related MBL polymorfism with MBL decreased functional activity has been reported to be protective against pre-eclampsia [15]. The level of MBL in the vaginal cavity changes during the menstrual cycle being produced locally by vaginal cells [16]. MBL seems to play an important role in embryo implantation since the analysis of uterine flushings, obtained at the time of oocyte retrieval for the in vitro fertilisation, revealed an increased level of MBL in patients with unexplained infertility compared with patients involved in IVF/ICSI for male or tubal infertility [17]. 

The aim of the present study was to evaluate the effect of sera obtained from pre-eclamptic patients on the process of vascular remodelling using in vitro models of trophoblast adhesion to and migration through DECs. We further investigate the ability of MBL to interfere with the process of trophoblast-endothelial cell interaction in order to define one possible mechanism responsible for the endovascular invasion failure in this severe multifactorial disease.

## 2. Material and Methods

### 2.1. Study Groups

In this study 11 pre-eclamptic and 11 normal pregnant women matched for gestation and parity were enrolled. The diagnosis of pre-eclampsia was established according to the standard criteria [18]. An informed consent was obtained from all women participating to the study. The study was approved by the Bioethical Committee of IRCCS, Burlo Garofolo, Trieste, Italy.

### 2.2. Collection and Processing of Sera and Measurement of MBL

Serum samples were obtained antepartum at the time of clinical diagnosis of the syndrome. The level of MBL in the sera was measured using the MBL oligomerELISA kit (Bioporto/Antibodyshop, Gentofte, Denmark).

### 2.3. Cell Isolation and Culture

EVT was purified from placental specimens after removal of decidual tissue and fetal membrane as previously described [3]. Briefly, placental tissue was incubated with HBSS containing 0.25% trypsin and 0.2 mg/mL DNase (Roche, Milan, Italy) for 20 min at 37°C. After fractionation through Percoll gradient, the leukocytes were totally removed by immunomagnetic beads coated with mAb to CD45 (Dynal, Invitrogen, Milan, Italy). EVT collected by negative selection were seeded in 25-cm^2^ flask coated with 5 *μ*g/cm^2^ fibronectin (FN, Roche), cultured overnight in RPMI 1640 (Gibco, Invitrogen), supplemented with 10% FCS, and finally detached by trypsin-EDTA treatment. The cells obtained under these conditions contained 95% cytokeratin 7-positive EVT and a few vimentin-positive stromal cells. The presence of contaminating leucocytes and ECs was excluded by RT-PCR assay for CD45 and FACS analysis with anti-vWF and anti-CD31 antibodies, respectively.

DECs were isolated from decidual biopsy specimens as previously described with some modifications [3]. Briefly, the tissue was finely minced, digested first with 0.25% trypsin (Sigma-Aldrich, Milan, Italy) and 50 *μ*g/mL DNase I (Roche) overnight at 4°C, and then with collagenase type 1 (3 mg/mL) (Worthington Biochemical Corporation, DBA, Milano, Italy) for 30 minutes at 37°C. The cells collected at the interface of Ficoll-Paque gradient (G&E Healthcare, Milan, Italy), after the centrifugation of the cell suspension at 690 ×g for 30 minutes, were positively selected with Dynabeads M-450 (Dynal, Invitrogen) coated with Ulex europaeus 1 lectin (Sigma-Aldrich). Cytofluorimetric analysis showed that more than 95% of the cells stained for vWF (Dako-Cytomation, Milan, Italy). The cells were seeded in 12.5-cm^2^ flask precoated with 5 *μ*g/cm^2^ fibronectin (Roche) and maintained in endothelial serum-free basal medium (GIBCO, Invitrogen) supplemented with 20 ng/mL bFGF (basic fibroblast growth factor) and 10 ng/mL EGF (epidermal growth factor) (GIBCO, Invitrogen).

### 2.4. In Vitro Immunofluorescence Analysis

EVT or DECs were plated on 8-chamber culture slides (BD Biosciences Discovery Labware, Milan, Italy) coated with FN (10 *μ*g/mL) at 37°C and left to adhere for 2 h. After fixation and permeabilization with FIX&PERM kit solutions (Invitrogen), EVT was stained with mAb OV-TL 12/30 anticytokeratin 7 (CK7, Dako-Cytomation, Milan, Italy), and DECs were stained with mAb BV9 antihuman VE-cadherin obtained through the courtesy of E. Dejana (Mario Negri Institute, Milan, Italy). The binding of these antibodies was revealed with goat antimouse FITC-conjugated secondary antibodies (Dako-Cytomation). Images were acquired using a Leica DM3000 microscope (Leica, Wetzlar, Germany) and the pictures were collected using a Leica DFC320 digital camera (Leica).

### 2.5. EVT Adhesion Assay

EVT adhesion assay was performed as previously described [19], with some modification. Briefly, 96-well plate was coated with 10 *μ*g/mL of C1q (Quidel, Medical Systems, Genoa, Italy), MBL (kindly provided by Prof. Peter Garred, Department of Clinical Immunology, Rigshospitalet, University of Copenhagen, Copenhagen, Denmark), or BSA (Sigma-Aldrich) in bicarbonate buffer at 4°C overnight and blocked with 1% BSA for 1 hour at room temperature. Cells were fluorescently tagged with the lipophilic dye DiI (Molecular Probes, Invitrogen), resuspended in RPMI with 0.1% BSA, and then added to protein-precoated wells. Labelled EVT cells previously incubated with sera (1 : 50) or MBL (2 *μ*g/mL) were added to C1q-coated wells for 45 min at 37°C in an air/CO_2_ incubator. The number of adherent cells were counted with Infinite200 (ABS 544 nm, EM 590 nm) (TECAN Italia S.r.l.) with reference to a calibration curves established with increasing number of labeled EVT cells.

### 2.6. EVT/DEC Adhesion Assay

The assay to evaluate adhesion of EVT to DECs has been previously described [3]. To evaluate the effect of sera or MBL on the adherence of trophoblast to DECs, DECs grown to confluence on 96-microwell plates (Costar, Milan, Italy) were cocultured for 45 min at 37°C in an air/CO_2_ incubator with an EVT cell suspension (10^5^ cells/100 *μ*L) labelled with a fluorescent dye (Fast DiI, Molecular Probes, Invitrogen) previously incubated with sera (1 : 50) or MBL (2 *μ*g/mL). The nonadherent EVT cells were removed by washing with Dulbecco-PBS containing Ca^2+^ and Mg^2+^ (0.7 mM). The number of adherent cells were counted with Infinite200 (ABS 544 nm, EM 590 nm) (TECAN Italia S.r.l., Milano, Italy) with reference to a calibration curves established with increasing number of labeled EVT cells.

### 2.7. Transendothelial Migration Assay

DECs (2 × 10^4^) were seeded onto 20 *μ*g/mL fibronectin-coated polycarbonate insert of a 24-well FloroBlock Transwell system (6.5 mm diameter, 8-*μ*m pores; BD Falcon) and used 5 days after plating [3]. EVT cell suspension (10^5^ cells/100 *μ*L) labelled with Fast DiI was added to the upper compartment of the transwell in the presence of sera (1 : 50) or MBL (2 *μ*g/mL). EVT cells were allowed to migrate for 24 h in human endothelial serum-free medium supplemented with basic FGF, recombinant EGF (Gibco Invitrogen). Cells present in the lower chamber or adherent to the lower surface of the transwell insert were counted with Infinite200 (ABS 544 nm, EM 590 nm) (TECAN Italia S.r.l.) with multiple reads of same well and the number of migrated cells were expressed as percentage with reference to a calibration curves established with increasing number of labelled EVTs plated in the lower chamber.

### 2.8. MBL-Binding Assay

To evaluate the binding of MBL to EVT, 10^5^ freshly isolated EVT cells were seeded onto 20 *μ*g/mL fibronectin-coated 96-well plate. The cells were incubated with 0.1 *μ*g/mL purified recombinant MBL, kindly provided by Professor Peter Garred, for 2 h at room temperature. The binding of the protein to the cells was analyzed using a monoclonal mouse antihuman MBL antibody (clone HYB131-01, Bioporto/Antibodyshop) 5 *μ*g/mL, followed by AP-conjugated secondary antibodies (Sigma-Aldrich) 1 : 10000. The enzymatic reaction was developed with PNPP (p-nitrophenyl phosphate) (Sigma-Aldrich; 1 mg/mL) as substrate and read kinetically at 405 nm using a Titertek Multiskan ELISA reader (Flow Labs, Milano, Italy).

### 2.9. Statistical Analysis

Results are expressed as mean ± SD or as *box plot* graphs, in which the line in the middle of the box represents the median where the lower and the upper edges of the box are the 1st and 3rd quartile, respectively. Statistical significance was determined using Student's *t* test to compare two groups of data. Values of *P* = 0.05 or less were considered to be statistically significant.

## 3. Results and Discussion

### 3.1. Sera Obtained from PE Patients Affect Trophoblast-Endothelial Cell Interaction

**Figure 1:**
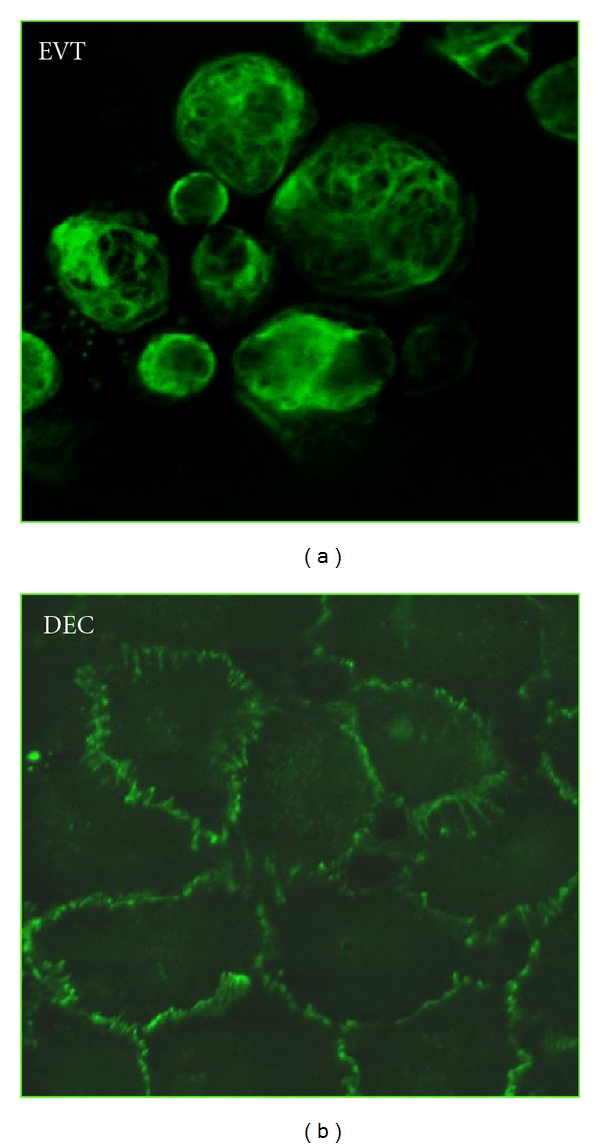
Immunofluorescence analysis of cells isolated from first trimester placenta. Purified EVTs were stained with mAb antihuman cytokeratin 7 (a) and DECs (b) with monoclonal anti-VE-cadherin. Images were acquired with Leica DM3000 microscope. Original magnification 100x.

**Figure 2:**
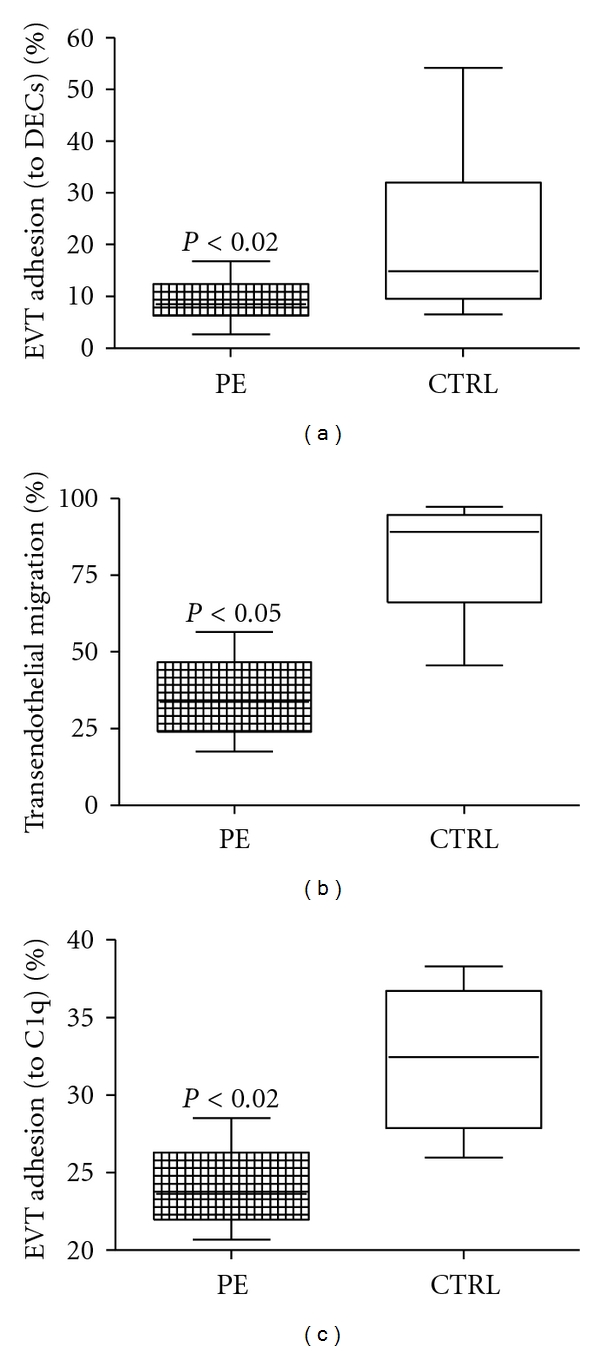
Effect of serum from pre-eclamptic and normal pregnant women in EVT/DEC interaction. Analysis of the effect of sera obtained from PE or normal women on the adhesion of EVTs to DECs (a), to C1q (c) and on transendothelial migration through DECs (b). EVTs were preincubated with PE or control sera (1 : 50). The results are expressed as percent of adhesion in reference to a standard curve. For each group, the line in the middle of the box represents the median. The lower and the upper edges of the box are the 1st and 3rd quartile, respectively.

We have previously demonstrated that trophoblast cells are able to adhere and migrate through endothelial cells [20]. A representative pattern of EVT cells and DECs used in the present investigation is shown in Figure 1. The effect of sera collected from PE and normal pregnant women at the same gestational age is presented in Figure 2(a). The results clearly show a significantly lower adhesion of EVT to DECs in the presence of pathological sera. The data were confirmed in at least three experiments using different preparation of trophoblast and endothelial cells. These results extend our previous observations obtained with sera from patients suffering from recurrent spontaneous abortion (RSA) [20]. These data suggest that endovascular invasion of trophoblast cells may be controlled by serum factors in several diseases associated with pregnancy failure. 

To further confirm the contribution of sera obtained from PE patients to the process of vascular remodelling, we next examined the ability of these sera to influence the migration of EVT through DECs. To this end, FastDiI-labelled EVT cells were allowed to migrate through to the monolayer of DECs grown to confluence on the insert of a transwell system in the presence of decidual conditioned medium as a source of chemotactic factors. Under these condition, the number of migrating EVT cells was approximately 70% (data not shown). The migration rate increased to approximately 95% following the addition of sera obtained from normal pregnant women. Conversely, the sera from PE patients elicited a strong inhibitory effect and cell migration dropped to 35% (Figure 2(b)).

Since C1q synthesized by DECs during pregnancy acts as a physical link between endovascular trophoblast and DECs favouring the process of vascular remodelling [4], we decided to investigate the effect of serum from PE patients and control pregnant women. The results presented in Figure 2(c) show that the sera obtained from PE patients significantly reduce the adhesion of EVT cells to C1q.

### 3.2. MBL Present in PE Sera Is Responsible for the Failure of the Interaction of EVT to DECs

MBL is one of the complement components that undergoes changes in pre-eclampsia. Than et al. [13] have published data indicating that patients had higher levels of plasma MBL compared to normal pregnant women. We, therefore, wondered whether MBL may be one of the factors responsible for the serum effect on EVT cell interaction with endothelial cells. 

**Figure 3:**
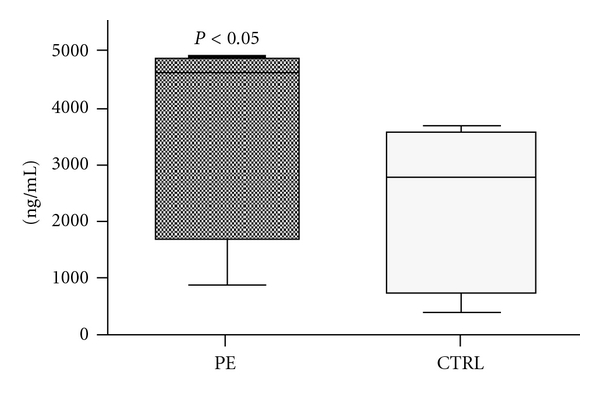
Analysis of serum levels of MBL in pre-eclamptic and control women. The level of oligomerized MBL in sera was measured using an MBL oligomer ELISA kit. The results are expressed as *box plot* graphs, in which the line in the middle of the box represents the median; the lower and the upper edges of the box are the 1st and 3rd quartile, respectively.

We initially evaluated the MBL concentration of sera from PE patients and normal pregnant women used in the inhibition experiments. The data shown in Figure 3 clearly indicate that PE patients had a significantly higher level of serum MBL than control women, confirming the observation by Than et al. [13]. The increase in MBL in normal pregnancy with respect to the level found in nonpregnant women [14] and the further increase in MBL concentration in patients with pre-eclampsia, which is considered an inflammatory condition [21], is in line with the fact that MBL acts as an acute phase protein [22].

**Figure 4:**
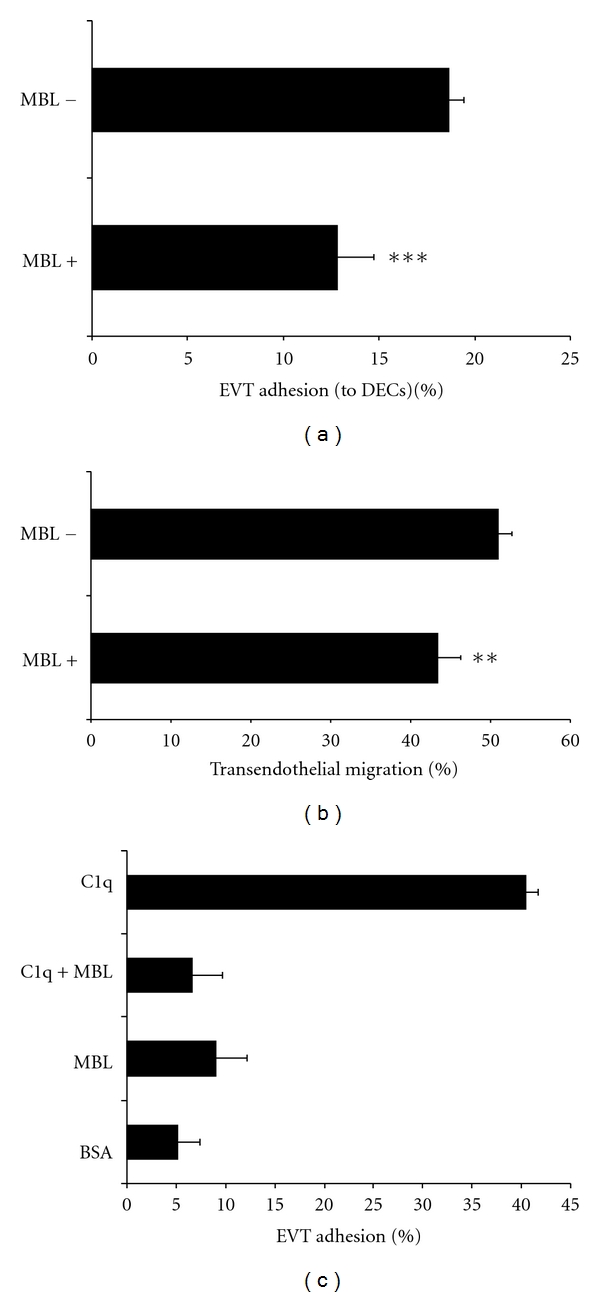
Analysis of MBL effect in the interaction between EVTs to DECs. To investigate the effect of MBL in the interaction between EVTs and DECs we performed adhesion (a) and migration assays through DECs, (b) in the presence of 2 *μ*g/mL of MBL. The graph in (c) confirm the ability of EVT to adhere to C1q, while MBL was not able to promote adhesion of EVT as compared to BSA used as negative control. The ability of MBL to inhibit the adhesion process was also evaluated on the binding of EVT to C1q (C1q + MBL). **P < 0.01; ***P < 0.001; ^§^ *P* < 0.00001.

To investigate the effect of MBL in the interaction between EVT and DECs, EVT were incubated with 2 *μ*g/mL recombinant MBL protein for 15 min at 37°C prior to be tested in the adhesion and migration assays performed as described above. As shown in Figure 4, both adhesion to and migration of EVT through DECs were significant lower in the presence of MBL. The role of MBL was evaluated also in EVT-C1q interaction. The data represented in Figure 4(c) confirmed the ability of EVT to adhere to C1q. MBL failed to promote adhesion of EVT, compared to BSA used as negative control but was able to substantially inhibit the adhesion of EVT to C1q. 

The binding site for MBL on EVT is still unknown. We have collected evidence indicating that MBL binds strongly to EVT (data not shown) and probably covers the binding site for C1q. The gC1q/p33 receptor for the globular head of C1q previously shown to be expressed on the surface of EVT [4] is unlikely to be involved in MBL-mediated inhibition of EVT-C1q interaction, since it is not an MBL receptor [23]. The only putative receptors for MBL are calreticulin and complement receptor-1 that bind to the tail domains of C1q and function also as receptors for MBL [23, 24]. These receptors, however, are present on ECs and interact with both MBL and C1q which compete with each other for their binding to the surface of ECs as shown by Oroszlàn et al. in cross-inhibition experiments [25]. Since DECs already express C1q on their surface, we postulate that MBL is probably unable to interact with EC membrane, though these data need to be confirmed.

## 4. Conclusion

In this study, we have shown that sera obtained from PE patients strongly inhibit the interaction between EVT and DECs, thereby controlling endovascular invasion of trophoblast cells, a fundamental process for the progression of pregnancy. MBL, one of the complement components shown to be present at increased levels in PE patient sera, is responsible for the inhibition of EVT adhesion to and migration through DECs. Our data also show that MBL interferes with the interaction of EVT with C1q expressed on DECs acting as a molecular bridge between endovascular trophoblast and DECs. The inhibitory effect of MBL on EVT adhesion to DECs is apparently unrelated to the ability of this complement component to activate the lectin pathway based on the observation that the levels of MBL-MASP2 complexes, which represent activation products of this pathway, are essentially similar in PE and control groups, indicating that the lectin pathway activation plays only a minor role in complement activation during pre-eclampsia [12]. On the basis of our results, we suggest that MBL, which is present at increased level in PE patients, may be one of the factors interfering with the endovascular invasion of trophoblast cells. Overall these data further indicate that proteins belonging to the C system may play alternative roles promoting physiological processes and inducing tissue damage.
